# A kit formulation for the preparation of [^89^Zr]Zr(oxinate)_4_ for PET cell tracking: White blood cell labelling and comparison with [^111^In]In(oxinate)_3_

**DOI:** 10.1016/j.nucmedbio.2020.09.002

**Published:** 2020-09-15

**Authors:** Francis Man, Azalea A. Khan, Amaia Carrascal-Miniño, Philip J. Blower, Rafael T.M. de Rosales

**Affiliations:** School of Biomedical Engineering & Imaging Sciences, King’s College London, London SE1 7EH, UK

**Keywords:** Zirconium-89, Cell tracking, PET, Nanomedicine, CAR-T, Cell therapy

## Abstract

**Background:**

Advances in immunology and cell-based therapies are creating a need to track individual cell types, such as immune cells (neutrophils, eosinophils, chimeric antigen receptor (CAR) T cells, etc.) and stem cells. As the fate of administered cells remains largely unknown, nuclear imaging could determine the migration and survival of cells in patients. [^89^Zr]Zr(oxinate)_4_, or [^89^Zr]Zr-oxine, is a radiotracer for positron emission tomography (PET) that has been evaluated in preclinical models of cell tracking and could improve on [^111^In]In-oxine, the current gold standard radiotracer for cell tracking by scintigraphy and single-photon emission computed tomography (SPECT), because of the better sensitivity, spatial resolution and quantification of PET. However, a clinically usable formulation of [^89^Zr]Zr-oxine is lacking. This study demonstrates a 1-step procedure for preparing [^89^Zr] Zr-oxine and evaluates it against [^111^In]In-oxine in white blood cell (WBC) labelling.

**Methods:**

Commercial [^89^Zr]Zr-oxalate was added to a formulation containing oxine, a buffering agent, a base and a surfactant or organic solvent. WBC isolated from 10 human volunteers were radiolabelled with [^89^Zr]Zr-oxine following a clinical radiolabelling protocol. Labelling efficiency, cell viability, chemotaxis and DNA damage were evaluated *in vitro*, in an intra-individual comparison against [^111^In]In-oxine.

**Results:**

An optimised formulation of [^89^Zr]Zr-oxine containing oxine, polysorbate 80 and 4-(2-hydroxyethyl)-1-piperazineethanesulfonic acid (HEPES) was developed. This enabled 1-step radiolabelling of oxine with commercial [^89^Zr]Zr-oxalate (0.1–25 MBq) in 5 min and radiotracer stability for 1 week. WBC labelling efficiency was 48.7 ± 6.3%, compared to 89.1 ± 9.5% (*P* < 0.0001, *n* = 10) for [^111^In]In-oxine. Intracellular retention of ^89^Zr and cell viability after radiolabelling were comparable to ^111^In. There were no significant differences in leukocyte chemotaxis or DNA damage between [^89^Zr]Zr-oxine or [^111^In]In-oxine.

**Conclusions, advances in knowledge and implications for patient care:**

Our results demonstrate that [^89^Zr]Zr-oxine is a suitable PET alternative to [^111^In]In-oxine for WBC imaging. Our formulation allows rapid, stable, high-yield, single-step preparation of [^89^Zr]Zr-oxine from commercially available ^89^Zr. This will facilitate the clinical translation of cell tracking using [^89^Zr]Zr-oxine.

## 1 Background

Recent developments in immunology and cell-based therapies are creating a need to track the migration of individual cell types. For example, neutrophils and eosinophils in asthma and chronic obstructive pulmonary disease were shown to have different distribution patterns [1–4], and there is considerable interest in tracking T-cells [5–7] and dendritic cells [8] in cancer and auto-immune diseases, or stem cells in regenerative medicine [9]. There is an emerging consensus [10,11], ac-companied by recognition by drug regulators [12], that development and trials of cell-based therapies should be accompanied by methods to determine the location, survival, proliferation and differentiation of administered cells both in animal models and human subjects. Imaging the *in vivo* trafficking of cells radiolabelled prior to administration is a clinically acceptable, informative, non-invasive approach that can be used in human subjects, is not limited by depth and requires no biopsy. Gamma scintigraphy, and more recently single-photon emission computed tomography (SPECT), with autologous leukocytes labelled with gamma-emitting radionuclides (^111^In, ^99m^Tc) has been a routine part of nuclear medicine since the 1970s [13] to detect sites of infection and/or inflammation [14,15]. Further developments in cell-based therapies [9,16–19] will require detection of small lesions and low numbers of cells, as well as better quantification, all of which could be achieved by positron emission tomography (PET).

Several positron-emitting radionuclides and radiotracers have been evaluated for cell tracking. The short half-lives of ^68^Ga (68 min) [20] and ^18^F (110 min) allow tracking only over brief periods, with 2-deoxy-2-[^18^F]fluoro-D-glucose ([^18^F]FDG) in particular suffering from rapid efflux and variable labelling efficiencies [21–23]. ^64^Cu has a longer half-life (12.7 h) and can be efficiently incorporated into cells using lipophilic tracers [6,24–26], but also suffers from rapid efflux from labelled cells. Zirconium-89 (^89^Zr) is a long half-life positron emitter (t_1/2_ = 78.4 h, β^+^: 23%) that could meet the need for cell tracking over longer (7 days or more), more biologically relevant periods [27]. It is commercially available for clinical use and is now commonly used for PET imaging of monoclonal antibody distribution in humans [28]. A method for using zirconium-89 for cell labelling that is highly analogous to [^111^In] In-oxine, has recently emerged [29–31]. [^89^Zr]Zr-oxine has been shown to label various cell types including tumour cell lines [27,29,30], bone marrow and dendritic cells [31–33], therapeutic T cells [34–36], and stem cells [37] as well as liposomes [38,39]. Its use to date has been in preclinical contexts only.

One of the main obstacles to the use of [^89^Zr]Zr-oxine-labelled cells in the clinic is its cumbersome synthesis and the absence of a simple one-step formulation (“kit”), such as exists for [^99m^Tc]Tc-D,L-hexamethylene-propyleneamine oxime ([^99m^Tc]Tc-HMPAO, [^99m^Tc]Tc-exametazime). This greatly restricts its clinical translation and commercial appeal for routine use in clinical trials. Furthermore, while some studies have investigated the effect of [^89^Zr]Zr-oxine labelling on cell function [34,35], only one preclinical study has directly compared [^89^Zr]Zr-oxine to the gold standard [^111^In]In-oxine [30]. Here we present the first kit formulation, and a simple, good manufacturing practices (GMP)-compliant, clinically translatable protocol for using it for rapid, one-step preparation of [^89^Zr]Zr-oxine, [^64^Cu]Cu-oxine and [^68^Ga]Ga-oxine for radiopharmaceutical applications, which will greatly enhance access of hospitals to cell tracking by PET in clinical diagnosis and trials of cell-based therapy. We demonstrate its application in radiolabelling human white blood cells (WBC) with [^89^Zr]Zr-oxine following a clinical protocol and provide a direct, intra-individual comparison with [^111^In]In-oxine.

## 2 Materials and methods

Unless otherwise indicated, reagents were obtained from Sigma-Aldrich and used without further purification. 8-hydroxyquinoline was obtained in ACS reagent grade, >99% purity. No-carrier-added, GMP-grade zirconium-89 (>150 MBq/nmol; BV Cyclotron, VU Amsterdam, NL) was purchased from PerkinElmer as [^89^Zr]Zr-oxalate in 1 M oxalic acid. [^111^In]InCl_3_ in 0.1 M HCl was purchased from Curium, UK. [^68^Ga] GaCl_3_ was eluted with 0.1 M HCl from a TiO_2_-based ^68^Ge/^68^Ga generator (lGG-100, Eckert & Ziegler). Copper-64 was prepared by ^64^Ni(p,n)^64^Cu nuclear reaction on a CTl RDS 112 11 MeV cyclotron as previously described [40]. Hydroxyethylstarch (HES200/0.5) was obtained from Carbosynth. Polysorbate 80 was obtained from Alfa Aesar. pH values were measured with a SevenCompact (Mettler-Toledo, UK) pH metre. Chemotaxis plates (polycarbonate membrane, 3.2 mm diameter, 5 μm pore size; ChemoTx® #101-5) were obtained from Neuroprobe. *N*- formylmethionyl-leucyl-phenylalanine (fMLP) was obtained from Santa Cruz Biotechnology. Antibodies were obtained from Miltenyi Biotec unless otherwise specified.

### 2.1 Kit formulation and quality control (QC)

Step-by-step instructions for preparing the kit formulation of [^89^Zr] Zr-oxine are provided in the Supplementary Material. Briefly, a solution containing 8-hydroxyquinoline (oxine; final concentration 0.5 mg/mL), HEPES (final concentration 1 M) and polysorbate 80 (final concentration 1 mg/mL) in ultrapure H_2_O was adjusted to pH 7.9-8.0 with aqueous NaOH. The resulting solution was filtered through a 0.2 μm membrane (Millipore), dispensed in 100 μL aliquots in sterile glass vials and stored at RT in the dark or freeze-dried and reconstituted later with 0.1–1 mL H_2_O. Alternative formulations were obtained by using NaHCO_3_ instead of NaOH, varying the amount of polysorbate 80 or substituting polysorbate 80 with EtOH (5% final concentration). [^89^Zr]Zr-oxalate (0.5–25 MBq, provided at 1.0–1.5 MBq/μL on reference day) in 1 M oxalic acid was added to the formulation and left at RT for 5 min before use. [^89^Zr]ZrCl_4_ was obtained by loading commercial [^89^Zr]Zr-oxalate onto a Sep-Pak Light Plus QMA cartridge (Waters #WAT023525), washing the cartridge with 10 mL H_2_O, and eluting with 500 μL of 1 M HCl [41]. The commercial [^111^In]In-oxine product [42] contains 50 μg 8-hydroxyquinoline, 100 μg polysorbate 80, 6 mg HEPES and 7.5 mg NaCl in a volume of 1000 μL, at a pH of 6.5–7.5. To keep labelling volumes equal to the [^89^Zr]Zr-oxine formulation when using [^111^In]In-oxine, a 10× concentrated solution (adjusted to pH 7 with 10 M NaOH) was prepared, containing 50 μg 8-hydroxyquinoline, 100 μg polysorbate 80, 6 mg HEPES and 7.5 mg NaCl in a volume of 100 μL. ^111^In in 0.1 M HCl (40–60 μL, 20–25 MBq) was added and left for 15 min at RT.

Product formation was confirmed by radio thin-layer chromatography (radioTLC) on instant TLC (ITLC)-SG paper (Macherey-Nagel) or Whatman no.1 paper (GE Healthcare) using 100% ethyl acetate (EtOAc) as the mobile phase. ITLC plates were read using a Mini-Scan™ radioTLC linear scanner (LabLogic Systems) equipped with a β^+^ probe (LabLogic B-FC-3600). Radiochemical purity of the final product was calculated as the activity associated with the [^89^Zr]Zr-oxine peak as a percentage of the total detected activity on the chromatogram.

To study the recovery of radiotracer from the vial, 10 μL aliquots were taken immediately after addition of [^89^Zr]Zr-oxalate and after 15, 30, 60, 120 min, 24, 48, 72 and 168 h. The aliquots were gammacounted 7 days after addition of [^89^Zr]Zr-oxalate and percentage recovery determined as the counts in each sample divided by the counts in the sample taken immediately after addition. For stability studies, samples were left at room temperature (RT) in the dark and analysed by radioTLC over 7 days. A diluted kit was obtained by further adding 900 μL H_2_O 5 min after addition of [^89^Zr]Zr-oxalate to the kit formulation.

The partition coefficient (logD) of the [^89^Zr]Zr-oxine and [^111^In]In-oxine formulations was determined by adding 10 μL (approx. 1 MBq) of each formulation to 1 mL of a 50:50 presaturated mixture of either 1-octanol and water or 1-octanol and phosphate-buffered saline (PBS), then vortexing for 5 min. Phase separation was then accelerated by brief centrifugation. 100 μL were taken from each phase and gammacounted.

### 2.2 Cell isolation

White blood cell isolation was performed in accordance with guidelines for radiolabelling WBC with [^99m^Tc]Tc-exametazime and [^111^In]In-oxine [43,44]. Briefly, peripheral venous blood (50–55 mL) was collected from healthy, male (*n* = 5) and female (n = 5) donors aged 22–32, in anticoagulant citrate dextrose solution A (ACD-A) blood collection tubes (BD Vacutainer #366645) using 20G needles, on two separate occasions for each donor. Cell-free plasma (CFP) was obtained by centrifuging 10–15 mL blood at 2000g for 10 min. For WBC isolation, 45 mL blood was mixed with 7 mL of HES200/0.5 (10% wt./vol. in sterile saline) and centrifuged at 8g for 45 min at room temperature. Platelets were depleted by washing the WBC layer twice with Ca^2+^/Mg^2+^-free PBS (with 10 min centrifugation at 150g). The remaining cell pellet was re-suspended in 3 mL PBS for radiolabelling.

### 2.3 Cell labelling: Labelling efficiency (LE), retention, viability

To WBC (1.6–4.8 × 10^8^) resuspended in 3 mL PBS, 100 μL of optimised [^89^Zr]Zr(oxinate)_4_ formulation (50 μg oxine, 18–21 MBq ^89^Zr) or 100 μL of 10× concentrated [^111^In]In(oxinate)_3_ formulation (50 μg oxine, 18–24 MBq ^111^In) were added. Cells were incubated for 20 min at RT with gentle swirling every 5 min. As an additional control, an aliquot of WBC was incubated with PBS only. Cells were then diluted with 50 mL PBS and centrifuged at 200g for 10 min. Supernatants and cell pellets were measured in a dose calibrator (CRC-25R, Capintec). The cells were suspended in CFP or assay medium (RPMI-1640 supplemented with 1% human serum, 2 mM L-glutamine, 100 U/mL penicillin and 100 μg/mL streptomycin) for further experiments. Viability was assessed using the Trypan Blue dye exclusion method. Cell labelling efficiency (LE%) was calculated as:

$$LE(\%)=\frac{\;\text{activity}\;\text{of}\;\text{cell}\;\text{fraction}\;}{\;\text{activity}\;\text{of}\;\text{cell}\;\text{fraction}\;+\;\text{activity}\;\text{of}\;\text{combined}\;\text{supernants}\;}.$$

For radiotracer retention studies, radiolabelled WBC were suspended in autologous CFP, in triplicate in a 24-well plate and incubated at 37 °C. Cells were collected after 4 h or 24 h and viability was determined as above. Cells were diluted with PBS, centrifuged at 200*g* for 10 min, and supernatants and pellets were measured in a dose calibrator to determine retention using the formula above.

### 2.4 Chemotaxis assay

After radiolabelling, cell pellets were subjected to red blood cell (RBC) lysis by hypotonic shock. Cells were resuspended in 4.5 mL cold H_2_O for 30 s, after which isotonicity was restored by addition of 0.5 mL 10× PBS. Lysed RBC were removed by centrifugation and the remaining WBC were resuspended in assay medium at 3.5 × 10^6^ cells/mL. The bottom wells of a chemotaxis plate were filled with 30 μL of assay medium, with or without 10 nM fMLP. On the top wells, 20 μL of cell suspension were then plated in triplicate and incubated for 45 min at 37 °C. Remaining cells in the top wells were removed, replaced by 40 μL of 5 mM EDTA in PBS to detach cells adhering to the membrane and the plate was incubated for 30 min at 4 °C. The top wells were emptied, the plate was centrifuged at 150g for 5 min and the cells in the bottom wells were counted using a haemocytometer. The chemotaxis index (CI) was calculated by dividing the number ofWBC in the wells containing fMLP by the number of WBC in the wells containing medium only.

### 2.5 DNA damage

WBC labelled with [^89^Zr]Zr-oxine or [^111^In]In-oxine or treated with PBS only were suspended in assay medium, seeded onto poly-L-lysine-coated coverslips and incubated for 30 min at 37 °C. Fixation and staining for γH2AX were performed as previously described [35]. Briefly, the cells were fixed and permeabilised with 3.7% formalin, 0.5% Triton X-100 and 0.5% IGEPAL® CA-630 in PBS. Staining was performed with an anti-γH2AX (Ser139) mouse mAb (1:1600; JBW301, Merck #05-636) and goat anti-mouse AF488-IgG (1:500; Jackson ImmunoResearch #115-545-062), followed by Hoechst 33342 for nuclei staining. Slides were imaged on an Eclipse Ti-E confocal microscope (Nikon) with a Plan Apo VC 60× oil DIC N2 objective (Nikon). Ten sections (0.4 μm thickness) were imaged. At least 30 nuclei/slide were imaged (2 slides/treatment). Maximal intensity projections of z-stacks were made using ImageJ v1.51p (http://imagej.nih.gov/ij). Nuclei and γH2AX foci were counted using CellProfiler v3.1.9 (http://cellprofiler.org) to determine the average numbers of γH2AX foci per nucleus in each image.

### 2.6 Flow-assisted cell sorting (FACS) of^89^Zr-labelled WBC

^89^Zr-labelled WBC were stained (15 min at 4 °C) with a combination of CD45-APC-Vio770 (#130-110-773), CD3-PE (REA613, #130-113-701), CD14-APC (#130-110-578), CD19-PE-Vio770 (REA675, #130-114-173) and CD16-FITC (REA423, #130-113-954) antibodies to sort samples into neutrophils, eosinophils, monocytes, T cells and B cells, or CD45-APC-Vio770, CD2354a-FITC (REA175, #130-117-800) and CD41a-PE (REA386, #130-121-429) to sort red blood cells and platelets. Compensation settings were adjusted using beads (anti-REA MACS® Comp, #130-104-693). Samples were analysed and sorted on a FACS-Melody instrument (BD Biosciences) equipped with blue (488 nm), yellow-green (521 nm) and red (633 nm) lasers. For each cell type, a determined number of events was collected, and the fractions were gamma-counted together with activity standards to determine the absolute amount of activity per cell.

### 2.7 Statistical analysis

Each subject provided WBC on two separate occasions (at least 1 week apart), once for [^89^Zr]Zr-oxine and once for [^111^In]In-oxine labelling, enabling differences between groups to be evaluated by Student’s two-tailed, paired t-test or Wilcoxon’s matched pairs signed-rank test, as appropriate. When additional factors were considered, analysis was performed using a repeated-measures Mixed Model (MM) in Prism v8.2 (GraphPad Software Inc.), with Tukey’s correction for multiple pairwise comparisons unless otherwise specified. Exact significance values are reported in each figure.

## 3 Results

### 3.1 QC method development

**Fig. 1:**
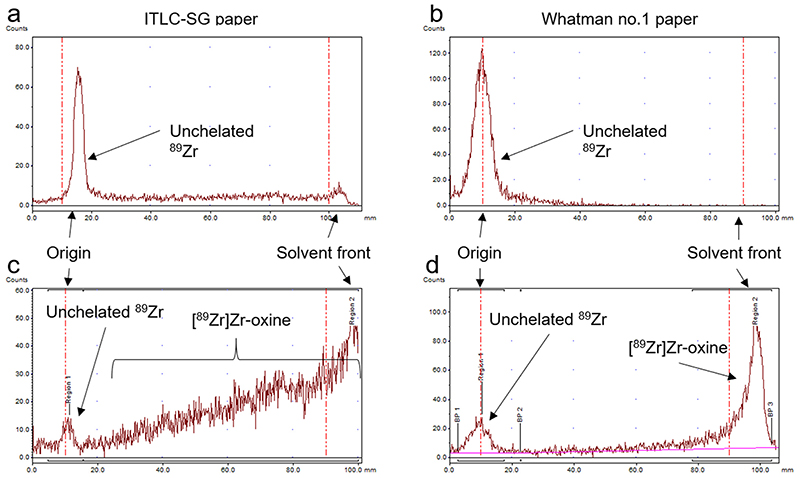
QC method for [^89^Zr]Zr-oxine. Representative radiochromatograms of [^89^Zr]Zr-oxalate in the absence of oxine (a, b) and [^89^Zr]Zr-oxine (c, d), spotted on ITLC-SG strips (a, c) or Whatman no.1 paper (b, d), with 100% EtOAc as mobile phase.

To evaluate the radiochemical yield of [^89^Zr]Zr-oxine, we optimised a simple radiopharmaceutical QC method. Initial measures by radioTLC on silica-gel impregnated glass fibre (ITLC-SG) using ethyl acetate showed no migration of unchelated ^89^Zr (R_f_ = 0, Fig. 1a), whereas [^89^Zr]Zr-oxine frequently showed marked streaking (Fig. 1c), possibly because the interaction of silanol groups with ^89^Zr leads to the dissociation of the metastable [^89^Zr]Zr-oxine complex during migration. Therefore, ITLC-SG is not an acceptable support for the QC of this radiotracer. In contrast, TLC on Whatman no. 1 paper shows a clear separation between unchelated ^89^Zr (R_f_ = 0, Fig. 1b) and [^89^Zr]Zr-oxine (R_f_ = 1, Fig. 1d). A strip length of 6 cm was found to provide clear separation between the two species, with a migration time of less than 8 min (Suppl. Fig. S1).

### 3.2 Kit formulation optimisation

**Fig. 2:**
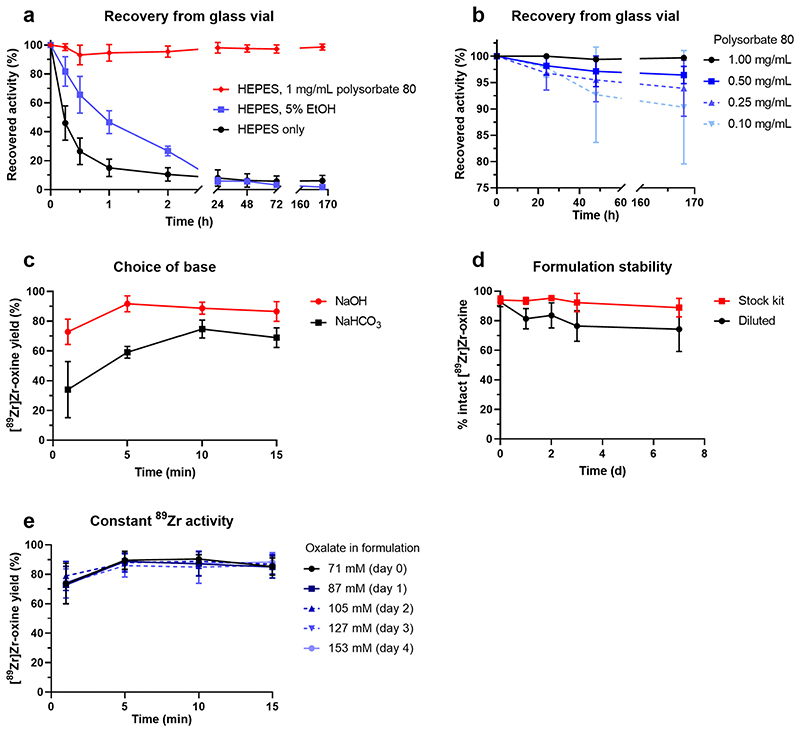
Formulation optimisation for [^89^Zr]Zr-oxine. (a, b) Recovery: percentage of [^89^Zr]Zr-oxine remaining in solution over time when formulated in HEPES buffer only and in presence of *5%* EtOHorvarying concentrations of polysorbate 80. Mean ± SDof *n* = 3 separate experiments. (c) Yield of [^89^Zr]Zr-oxine over time in HEPES buffers (pH 7.9) containing NaOH (*n* = 5) or NaHCO_3_ (*n* = 4), measured by radioTLC. (d) Stability: [^89^Zr]Zr-oxine as percentage oftotal^89^Zr activity in solution over 7 days, measured by radioTLC (n = 3), in original 100 pLformulation or after 10-fold dilution with H_2_O. (e) Formation rate and radiochemical yield of [^89^Zr]Zr-oxine as a function of oxalate content and ^89^Zr decay. [^89^Zr]Zr-oxalate in 1 M oxalic acid (1.1-1.5 MBq/μL on day 0, approximately 4-5 days after production in the cyclotron) was added to 20 μL aliquots of kit formulation on day of reception (day 0) and 4 subsequent days. To keep total ^89^Zr activity constant, increasing volumes of [^89^Zr]Zr-oxalate solution were added on each day, resulting in increasing concentrations of oxalate ions in the final product. Mean ± SD of n = 3 separate experiments.

**Fig. 3:**
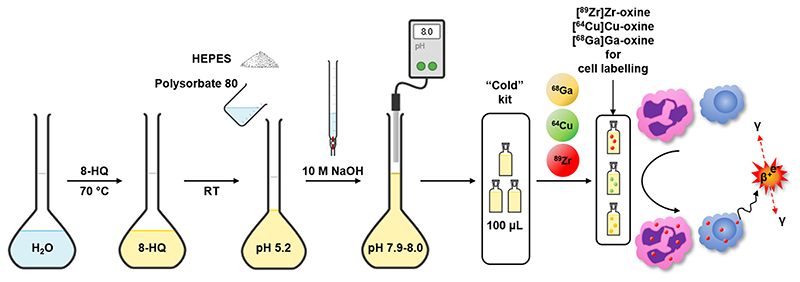
Preparation and use of the kit formulation for [^89^Zr]Zr-oxine, [^64^Cu]Cu-oxine and [^68^Ga]Ga-oxine. 8-hydroxyquinoline is dissolved in water, followed by addition of HEPES and polysorbate 80 and pH adjustment to 7.9-8.0 with concentrated NaOH. This solution can be divided into single-use aliquots, *e*.*g*. one patient dose, and radiolabelled with commercial zirconium-89, copper-64 or gallium-68 as and when required. Alternatively, a central radiopharmacy facility could provide the radiolabelled product to the site performing the cell radiolabelling. The formulated [^89^Zr]Zr-oxine (or [^64^Cu]Cu-oxine/[^68^Ga]Ga-oxine) can be used to radiolabel various cell types, such as granulocytes or T-cells, without any further steps, for PET imaging.

A kit formulation for [^89^Zr]Zr-oxine requires a base to neutralise the acidic solution of ^89^Zr supplied and a buffer to maintain the solution at pH 7–8. It was found that 100 μL of HEPES-buffered formulation (containing 50 μg 8-hydroxyquinoline and 52.5 μmol NaOH) was capable of buffering (pH ≥ 7.0) a maximum of 18 μL of [^89^Zr]Zr-oxalate solution (153 mM oxalate in final product). [^89^Zr]Zr-oxine formulated in 1 M HEPES buffer (pH 7.9) was found to rapidly adhere to glass vessels, with only 46% of the added activity recoverable from the vial 15 min after addition of [^89^Zr]Zr-oxalate. Including 5% EtOH in the solution delayed this phenomenon but did not prevent it, with 46% recoverable activity after 1 h and less than 6% after 24 h (Fig. 2a). In contrast, addition of 1 mg/mL polysorbate 80 prevented adhesion to the glass vessel and resulted in 98.7% recovery of activity up to 1 week after addition of [^89^Zr]Zr-oxalate (Fig. 2a, b). Reducing the concentration of polysorbate 80 resulted in minor losses of product. Replacing NaOH with NaHCO3 led to slower formation of [^89^Zr]Zr-oxine and reduced yields (Fig. 2c). The optimised formulation, a 100 μL solution at pH 7.9–8.0 containing 50 μg oxine, 1 M HEPES, NaOH and 1 mg/mL polysorbate 80, was stable for 7 days in concentrated format (Fig. 2d). The preparation and use of the kit formulation are schematically represented in Fig. 3. Diluting the formulation to 1 mL with H_2_O after addition of [^89^Zr]Zr-oxalate reduced the percentage of intact product from 92% to about 75% within 24–48 h. When using [^89^Zr]Zr-oxalate (1 Moxalic acid) as starting material, [^89^Zr] Zr-oxine was typically formed in 85–92% radiochemical yield (95% confidence interval, *n* = 15) 5 min after addition of [^89^Zr]Zr-oxalate (Fig. 2e). Increasing the volume of [^89^Zr]Zr-oxalate up to a final oxalate concentration of 153 mM to account for ^89^Zr decay did not affect the formation rate or final radiochemical yield of [^89^Zr]Zr-oxine (Fig. 2e). The percentage of “free”, unchelated oxine in a 20 MBq batch of [^89^Zr]Zr-oxine was calculated to be >99.86% (Supplementary Material, Table S1). The partition coefficient of the formulated [^89^Zr]Zr-oxine was found to be 0.80 ± 0.26 in water ([^111^In]In-oxine: 1.09 ± 0.03, *n* = 3, *P* = 0.18) and 0.60 ± 0.06 in PBS ([^111^In]In-oxine: 0.72 ±0.11, n = 3, *P* = 0.08; Suppl. Fig. S2).

As well as zirconium-89, the optimised oxine formulation was shown to form lipophilic complexes of oxine with gallium-68, copper-64 and indium-111 in high yield (Suppl. Fig. S3), and therefore may be useful for labelling cells or nanomedicines with these radionuclides.

### 3.3 Labelling efficiency and comparison with [^In^in]in-oxine

A detailed standard operating procedure for labelling WBCs with the kit formulation of [^89^Zr]Zr-oxine is provided in the Supplementary Material. Using the optimised kit, we evaluated the labelling of human WBCs with [^89^Zr]Zr-oxine, in comparison with [^111^In]In-oxine formulated similarly to the commercial product [42]. WBCs were obtained from 10 healthy donors, following a clinical protocol for WBC labelling [44], and an intra-individual comparison of labelling with [^89^Zr]Zr-oxine and [^111^In]In-oxine was performed. In this study we have attempted to replicate real-world conditions as closely as possible, rather than standardise every single parameter (*i*.*e*. by using the same number of WBCs from each donor) or splitting WBC batches for labelling with each radiotracer. Labelling with each radiotracer was performed on separate occasions for each donor to comply with local ethical limits regarding blood donations.

**Fig. 4:**
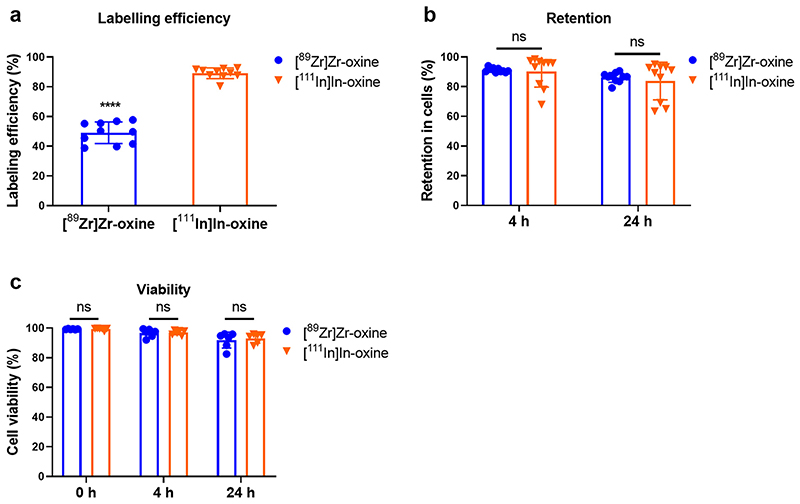
WBC radiolabelling with [^89^Zr]Zr-oxine and [^111^In]In-oxine. (a) WBC labelling efficiency with [^89^Zr]Zr-oxine and [^111^In]In-oxine. Mean ± SD of *n* = 10 per group. ****P < 0.0001 (Student’s two-tailed paired t-test). (b) Retention of^89^Zrand ^111^In in WBC, 4 h and 24 h after radiolabelling. n = 10 per group, *P >* 0.05 (Wilcoxon’s matched pair signed-rank test). (c) Viability of ^89^Zr- and ^111^In-labelled WBC after 4 h and 24 h. Mean ± SD of *n* = 6 per group, Student’s two-tailed paired *t*-test.

The labelling efficiency of WBCs with [^89^Zr]Zr-oxine was 48.7 ± 6.3%, *c.f.* 89.1 ± 9.5% (P <0.0001, *n* = 10) for [^111^In]In-oxine (Fig. 4a), resulting in an average activity of 32.9 ± 9.2 kBq/10^6^ cells for [^89^Zr]Zr-oxine and 58.2 ± 26.1 kBq/10^6^ cells for [^111^In]In-oxine. Cellular retention of ^89^Zr was 91.4 ± 1.4% after 4 h incubation in autologous CFP and 86.6 ± 2.9% after 24 h (Fig. 4b), and cell viability was 99.4 ± 0.3% immediately after labelling, 97.0 ± 2.3% after 4 h and 92.6 ± 4.8% after 24 h (Fig. 4c). There were no significant differences in retention or viability between [^89^Zr]Zr-oxine and [^111^In]In-oxine (P > 0.05, *n* = 6–10). Visual inspection of the cell preparations revealed no clumping or cells, aggregation or presence of fibrin clots.

**Fig. 5:**
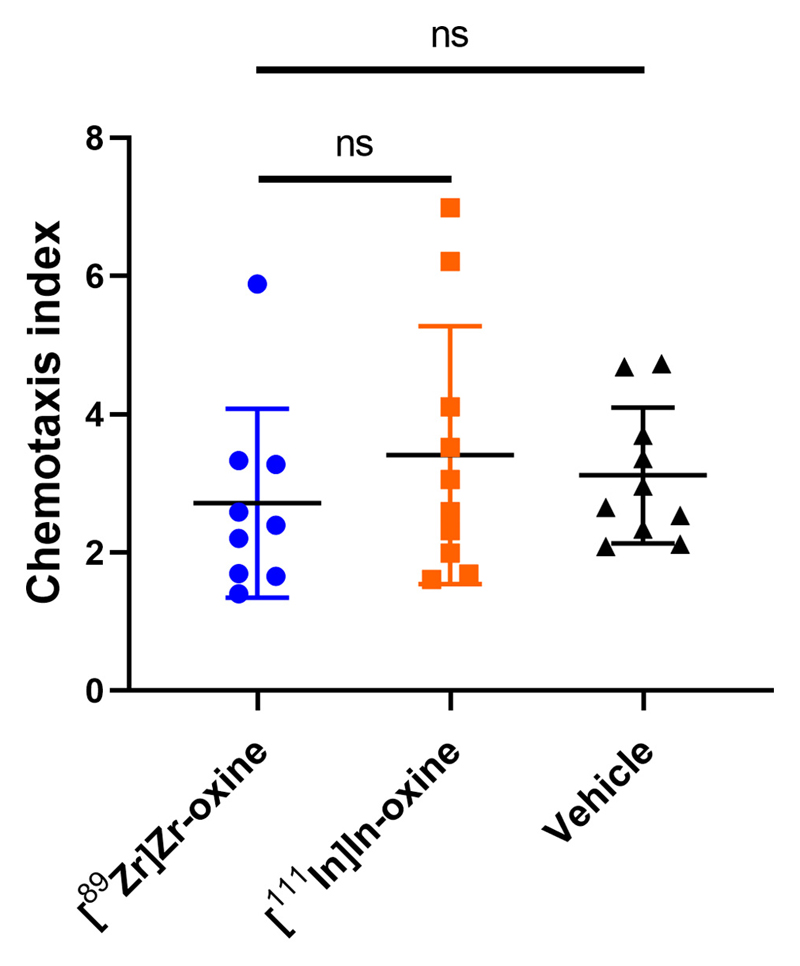
Chemotaxis of radiolabelled WBC. Cells were incubated with [^89^Zr]Zr-oxine, [^111^In]In-oxine, or vehicle only. The chemotaxis index is the number of cells migrating towards 10 nM fMLP divided by the number of cells migrating towards vehicle, averaged from triplicates for each sample. Each point represents one donor. Bars represent the mean ± SD of *n* = 9, 10 and 10 individual samples. P ≥ 0.05 for all comparisons (mixed-effect analysis with Tukey’s correction for multiple comparisons).

The functionality of radiolabelled WBCs was tested using an *in vitro* chemotaxis assay, where the number of WBCs migrating in response to 10 nM fMLP was measured. The chemotaxis index of ^89^Zr-labelled WBCs was 2.7 ± 1.4 (n = 9), *c.f.* 3.4 ± 1.9 (n = 10) for ^111^In-labelled WBCs and 3.0 ± 1.0 (n = 10) for non-labelled WBCs (Fig. 5). There were no significant differences between the groups. Importantly, the chemotaxis indexes were all >1, demonstrating an active migration towards fMLP. There was no apparent trend relating the amount of activity per cell and the chemotaxis index (Suppl. Fig. S4).

**Fig. 6:**
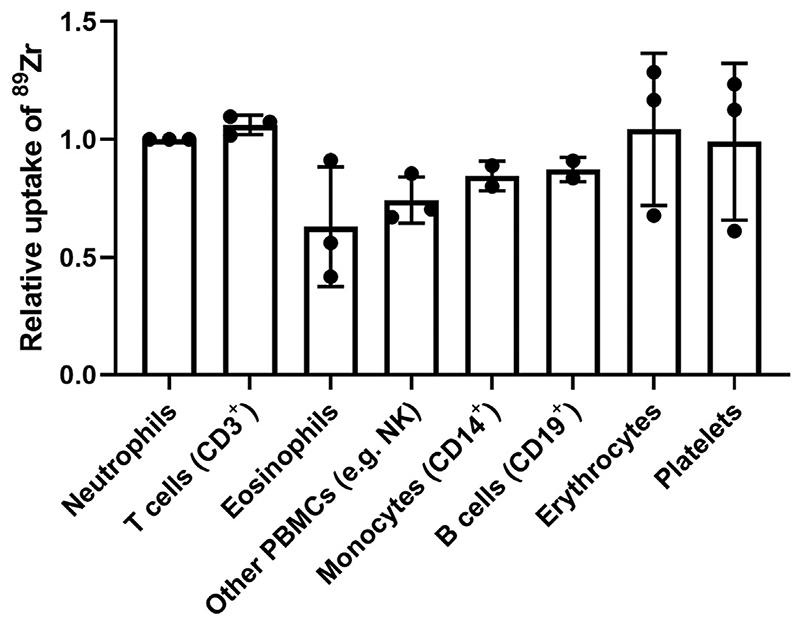
Relative uptake of ^89^Zr per cell in WBC populations after labelling mixed WBC with [^89^Zr]Zr-oxine. Radiolabelled WBC were sorted by FACS and gamma-counted to determine the absolute activity per cell (>10,000 cells/sample). Data are expressed as a ratio of the activity per cell in the neutrophil population of each respective donor. Each point represents cells from an individual donor, bars represent the mean value of *n* = 2 or 3 donors.

To determine whether certain subtypes of WBC had preferential uptake of [^89^Zr]Zr-oxine, radiolabelled WBC were stained with fluorescent monoclonal antibodies, automatically sorted and gamma-counted. For practical reasons, only a small fraction of the radiolabelled cells was sorted. There were large differences in average activity per cell (range 1–4 kBq/10^6^ cells, Suppl. Fig. S5) between donors despite comparable labelling efficiencies (fraction of activity in cell pellet) because a fixed patient dose of [^89^Zr]Zr-oxine (20 MBq) was used whereas the number of WBCs isolated from each donor was highly variable. Results are therefore expressed as relative uptake of ^89^Zr per cell in each population (Fig. 6), taking neutrophils as reference (1.00). The relative activity of ^89^Zr per cell was 1.06 ± 0.04 in lymphocytes, 0.63 ± 0.25 in eosinophils, 0.74 ± 0.10 in NK cells, 0.87 ± 0.05 in B cells, 0.85 ± 0.06 in monocytes, 0.99 ± 0.33 in platelets and 1.04 ± 0.32 in erythrocytes.

**Fig. 7:**
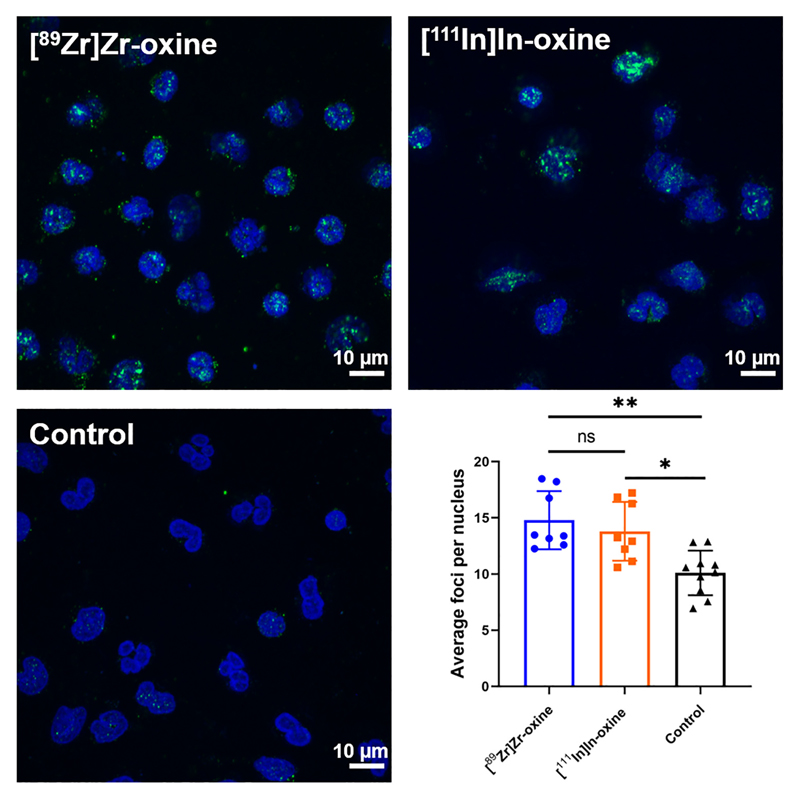
Comparison of DNA damage induced by [^89^Zr]Zr-oxine and [^111^In]In-oxine in WBC. Representative images (maximum intensity projections) of WBC labelled with [^89^Zr]Zr-oxine (*top left*), [^111^In]In-oxine (*top right*) or PBS only (bottom left), showing γH2AXfoci (green) and cell nuclei (blue). Scale bars represent 10 μm. *Bottom right*: average number of γH2AX foci per nucleus (at least 30 nuclei analysed per sample). Each point represents an individual donor, bars show the mean value of *n* = 8 donors. ns: P ≥ 0.05, *P < 0.05, **P < 0.01 (mixed-effect analysis with Tukey’s correction for multiple comparisons, performed on log-transformed data).

The effect of radiolabelling on DNA damage, assessed in terms of double-strand break formation, was determined by counting the number of γH2AX foci present in individual WBCs. The number of foci per nucleus was 14.8 ± 2.6 for [^89^Zr]Zr-oxine, 13.8 ± 2.6 for [^111^In]In-oxine and 10.1 ± 2.0 for unlabelled cells (Fig. 7). The differences between radiolabelled and non-radiolabelled samples were significant for both [^89^Zr]Zr-oxine and [^111^In]In-oxine, however there was no statistical difference between the two radiotracers. There was no apparent trend relating the number of foci per nucleus and the activity per cell after radiolabelling (Suppl. Fig. S6).

## 4 Discussion

[^89^Zr]Zr-oxine was previously shown to be a useful cell labelling agent for PET imaging in animal models by our group and others [30–36]. In the clinic, the radiolabelling of mixed WBCs for infection imaging is one of the most common application of cell labelling, with the greatest collective experience within the nuclear medicine community. For the clinical translation of this radiotracer, we believe it is crucial to (a) simplify its synthesis to the point that it is usable with as few steps as possible by radiopharmacy operators, and (b) perform a direct comparison with the gold standard [^111^In]In-oxine in the clinically relevant model of radiolabelled WBCs.

Our previous synthesis of [^89^Zr]Zr-oxine from [^89^Zr]Zr-oxalate involved the use of chloroform as a solvent and subsequent evaporation and redissolution in a small amount of dimethyl sulfoxide or ethanol [29]. While relatively simple from a research perspective, this is far from ideal for the clinic as it involves several steps, requires meticulous precision during the neutralisation step and involves organic solvents (requiring further QC tests) and solvent evaporation, with an overall radiochemical yield of 60–80%. Sato et al. describe the synthesis from [^89^Zr]ZrCl_4_ [31], requiring prior conversion of the less-reactive [^89^Zr] Zr-oxalate [41] in which form ^89^Zr is typically produced [45,46] and available commercially. Leaving the conversion step to the final user results in additional manipulation, time and operator exposure, and lower activity concentration. Indeed, about 350 μLof1M hydrochloric acid are required to extract 80–90% of the [^89^Zr]Zr^4+^ from a typical anionexchange cartridge. Considering the typical batches (50–200 MBq) available commercially, this results in a large volume (35–200 μL) of 1 M hydrochloric acid to neutralise, requiring further dilution to reach isotonicity. This would result in final labelling volumes larger than recommended for WBC [44], leading to low labelling efficiencies. Furthermore, the amounts of oxine and polysorbate 80 described in that method are unnecessarily high and the process as described requires several steps. Weist et al. used unprocessed [^89^Zr]Zr-oxalate and a 1 M HEPES buffer, in a 2-step process [34]. However, we have demonstrated in the present work that [^89^Zr]Zr-oxine rapidly adheres to reaction vessels in the absence of surfactant. Socan et al. have described the on-cartridge preparation of oxine complexes of several radiometals, but reported only 64% RCY of [^89^Zr]Zr-oxine after 2 h, including an extraction step using n-octanol [47]. In summary, none of the published methods are suitable for clinical radiopharmacy applications. Here we present a formulation that allows rapid, stable, high-yield (≥85%) preparation of [^89^Zr]Zr-oxine in a single step, without further processing of commercially available zirconium-89. The commercial formulation of [^111^In]In-oxine [42] is or was provided in 1 mL units. In comparison, [^89^Zr]Zr-oxine is formulated in 100 μL units, with a 10× higher concentration of oxine and polysorbate 80 to ensure formulation stability and a 40× higher concentration of HEPES to buffer the solution because current production methods require a large amount of acid to extract [^89^Zr]Zr^4+^ from the bombardment target. Our formulation of [^89^Zr]Zr-oxine therefore contains the same total amount of oxine and polysorbate 80 as the [^111^In]In-oxine formulation, which should facilitate regulatory approval, but in a 10× lower volume. Oxalic acid is rapidly neutralised by NaOH, 1 M HEPES ensures pH buffering, and the amount of polysorbate 80 is optimised to ensure stability of the radiotracer for up to 1 week. In practice, this allows the end-user to prepare [^89^Zr]Zr-oxine in a single manipulation simply by transferring [^89^Zr]Zr-oxalate from its delivery vial, without modification, to an off-the-shelf vial (“kit”) of oxine formulation. Alternatively, it can be used as a basis for the shipping of ready-to-use, single- or multiplepatient doses of [^89^Zr]Zr-oxine from a central site to distant scanning centres, as has been the case previously for [^111^In]In-oxine. The radiolabelling agent thus prepared can be added directly to a cell suspension in a procedure analogous to that used conventionally for ^111^In-labelling using [^111^In]In-oxine, as described in the Supplementary Material. In line with conventional radiolabelling protocols, the washing step after radiolabelling ensures the amount of free oxine and unchelated zirconium-89 in the final administered product is further reduced. Sterility and endotoxin testing can be performed according to local regulations.

The labelling of WBC with [^89^Zr]Zr-oxine was reliable, consistently achieving 45–50% labelling efficiency with 160–480 million cells. This is significantly lower than that achieved with [^111^In]In-oxine in the same conditions. As the formulations of [^89^Zr]Zr-oxine and [^111^In]In-oxine both contain the same total amount of oxine, in large excess (3 orders of magnitude) compared to the amount of the respective radiometals (see Supplementary Material, Table S1), it unlikely that the difference in radiolabelling efficiencies can be explained by differences in amounts of unchelated oxine. Instead, we suggest the difference is most likely due to the physicochemical properties of each radiotracer. The partition coefficients of [^89^Zr]Zr-oxine in water and PBS were not significantly different from those of [^111^In]In-oxine, suggesting other factors are involved, such as potentially slower intracellular dissociation kinetics of [^89^Zr]Zr-oxine compared to [^111^In]In-oxine. This may lead to increased leakage of ^89^Zr from cells over time, and consequently increased “parasite” signal on PET scans, although in the present study we did not observe major losses nor significant differences in retention compared to [^111^In]In-oxine *in vitro* over 24 h. A previous study comparing [^89^Zr]Zr-oxine and [^111^In]In-oxine in tumour cell lines suggested the retention of ^89^Zr was higher than that of ^111^In after 24 h [30]. lf imaging cells at later time points (> 24 h) is desired, then longer radiotracer retention studies are warranted. In studies with T-cells, bone marrow cells or stem cells [32,34,35,37], significant leakage (30–60%) of [^89^Zr]Zr-oxine was observed over 2–3 days *in vitro*, with the rate of leakage slowing down after 3 days. However, preclinical PET images from the same studies suggest better *in vivo* retention of ^89^Zr than *in vitro*, and it has been shown that PET images can be improved by administering deferoxamine intravenously to accelerate the renal clearance of ^89^Zr released from radiolabelled cells [48].

Using the typical patient dose of [^111^In]In-oxine (approx. 20 MBq) as reference, we aimed for patient doses of [^89^Zr]Zr-oxine of about 9–10 MBq. Considering the sensitivity of current-generation clinical PET and SPECT scanners and the relatively low positron yield of ^89^Zr (23%), we estimate that 9–10 MBq of ^89^Zr should result in a 5-to-10-fold increase in useful counts compared to 20 MBq of ^111^In and therefore lead to improved image quality. Another advantage of ^89^Zr over ^111^In is that PET/CT facilitates signal quantification and will allow better determination of the cell numbers reaching the target organ. Sato et al. have recently performed dosimetry studies of ^89^Zr-labelled NK cells in non-human primates [48], suggesting such amounts of activity to be safe for the liver and spleen. Furthermore, their study showed good quality images in a clinical PET/CT scanner with ^89^Zr activities in the range of 13–44 kBq/10^6^ cells, similar to the average activity of 32.9 kBq/10^6^ cells achieved in the present study. Crucially, cell viability and retention of ^89^Zr in cells in the presence of plasma were high and comparable to those of ^111^In, suggesting that radiotracer leakage will be low after intravenous administration and that the PET signal will accurately reflect the location of intact cells, as previously observed in pre-clinical studies [35]. We did not perform *in vivo* experiments as there is no justification in this case for using unpurified human leukocytes, *i*.*e*. predominantly neutrophils, in a small animal model. Our results using flow-assisted cell sorting indicate no preferential uptake by any specific leukocyte population. In contrast, it has been shown previously that [^99m^Tc]Tc-HMPAO accumulated preferentially in eosinophils [1], with the clinical implication that [^99m^Tc]Tc-HMPAO WBC scans disproportionately represent the distribution of eosinophils. Our results suggest this phenomenon is not expected with [^89^Zr]Zr-oxine. However, because there is significant uptake in RBCs and platelets, better separation of these cells from WBC (*e*.*g*. using automated cell separators) will improve signal specificity and target-to-background ratio.

Leukocyte chemotaxis towards pathogens and inflammatory stimuli is the biological basis for WBC imaging. We found that ^89^Zr-labelled WBC retained their chemotactic properties *in vitro*, with no significant differences compared to [^111^In]In-oxine and unlabelled control cells. This suggests that radiolabelling with [^89^Zr]Zr-oxine will not affect *in vivo* properties of administered WBC at least within a few hours following radiolabelling and will provide clinically useful images. While both radiotracers resulted in significantly higher DNA damage compared to non-radiolabelled cells, no differences were observed between the radiotracers. Notably, our results were strengthened by the intraindividual nature of the comparison between [^89^Zr]Zr-oxine and [^111^In]In-oxine.

Considering the translational aspect of this technique, we also emphasise that WBC labelling was performed following the guidelines established for [^111^In]In-oxine and [^99m^Tc]Tc-HMPAO [43,44], and thus requires no modifications from existing protocols beyond the provision of additional shielding for ^89^Zr.

## 5 Conclusions

In summary, we provide a clinically applicable, kit-based method for the simple and reliable preparation of ready-to-use [^89^Zr]Zr-oxine, in a single step, from commercially supplied [^89^Zr]Zr-oxalate and demonstrate its use in radiolabelling WBC. Our work further demonstrates that, from a cell labelling perspective, [^89^Zr]Zr-oxine is an appropriate PET equivalent of [^111^In]In-oxine, and should be investigated in the clinic not only for WBC labelling in infection diagnosis, but potentially also for upcoming trials of cell-based therapies such as CAR-T and stem cells.

## Supplementary Material

Supplementary material

## Abbreviations

[^18^F]FDG: 2-deoxy-2–[^18^F]fluoro-D-glucose
[^99^mTc]Tc-HMPAO Technetium: (^99m^Tc) hexamethylpropyleneamine oxime
CAR-T: chimeric antigen receptor T cell(s)
CFP: cell–free plasma
DNA: deoxyribonucleic acid
EDTA: ethylenediaminetetraacetic acid
EtOH: ethanol
FACS: flow–assisted cell sorting
fMLP: *N*–formyl–Met–Leu–Phe
GMP: good manufacturing practice
HEPES: 4–(2–hydroxyethyl)–1–piperazineethanesulfonic acid
HES: hydroxyethyl starch
ITLC: instant thin-layer chromatography
LE: labelling efficiency
PBS: phosphate-buffered saline
PET: positron emission tomography
QC: quality control
radioTLC: radio-thin-layer chromatography
RBC: red blood cell(s)
RT: room temperature
SD: standard deviation
SPECT: single-photon emission computed tomography
WBC: white blood cell(s)
γH2AX: gamma H2A histone family member X
