# Supplementary material for: A kit formulation for the preparation of [89Zr]Zr(oxinate)4 for PET cell tracking: White blood cell labelling and comparison with [111In]In(oxinate)3
Source: Nucl Med Biol. Author manuscript; Available in PMC 2021 Feb 16. (PMC7116765; doi:10.1016/j.nucmedbio.2020.09.002)
Supplement: Supplementary material [file EMS115486-supplement-Supplementary_material.docx]

**A kit formulation for the preparation of [^89^Zr]Zr(oxinate)_4_ for PET cell tracking: White blood cell labelling and comparison with [^111^In]In(oxinate)_3_**

Francis Man^1^*, Azalea Khan^1^, Amaia Minino^1^, Philip J. Blower^1^, Rafael T. M. de Rosales^1^*

**Supplementary Material**

**Protocol for the preparation of a kit formulation of [^89^Zr]Zr(oxinate)_4_ ([^89^Zr]Zr-oxine) and radiolabelling white blood cells**

NB: It is assumed that operators have appropriate training and procedures in place for work with high-energy gamma- and positron-emitting radionuclides such as ^89^Zr.

1. Kit preparation:
   1. Reagents, materials:
      1. 8-hydroxyquinoline (oxine), CAS 148-24-3
      2. 4-(2-hydroxyethyl)-1-piperazineethanesulfonic acid (HEPES), CAS 7365-45-9
      3. Sodium hydroxide (NaOH), CAS 1310-73-2
      4. Polysorbate 80 (Tween® 80), CAS 9005-65-6
      5. Water (Ultra-pure)
      6. ^89^Zr solution (typically in 1 M oxalic acid or 1 M hydrochloric acid)
      7. Ethyl acetate (EtOAc), CAS 141-78-6
      8. Whatman® no.1 paper, in 1 × 8 cm strips
      9. Electronic pH-meter
   2. Prepare solutions A and B:
      1. Solution A: 10 M NaOH (CAUTION: corrosive, exothermic). Weigh 20 g of NaOH and place in a 50 mL volumetric flask. Add approximately 35 mL of ultrapure water and gently swirl until dissolved. Add water to the 50 mL mark.
      2. Solution B: 10 mg/mL polysorbate 80. Weigh 500 mg of polysorbate 80 and place in a 50 mL volumetric flask. Add approximately 35 mL of ultrapure water and gently swirl until dissolved. Add water to the 50 mL mark.
   3. Weigh out 50 mg of 8-hydroxyquinoline, place in a 100 mL volumetric flask. Add approximately 70 mL of ultrapure water to the volumetric flask.
   4. Heat the flask to 70-80 °C in a water bath for 10 min, shaking the flask to aid dissolution. Cool down to room temperature.
   5. Add 23.83 g of HEPES and swirl until dissolved.
   6. Add 10 mL of solution B.
   7. Add solution A (approximately 5.25 mL) until the pH reaches 7.9-8.
   8. Add ultrapure water to the 100 mL mark and invert to homogenise.
   9. (optional) Sterile-filter the resulting solution through a 0.2 µm PVDF or PES membrane.
   10. (optional) Divide the filtered solution into 100 µL aliquots.

Notes:

- - 1. The solution can be kept at room temperature in the dark for at least 6 months.
    2. The solution can be autoclaved if required.

1. Radiotracer preparation and quality control (QC)
   1. To 100 µL of kit formulation, add a maximum of 18 µL of ^89^Zr solution
   2. Briefly mix by swirling and leave for 5 min at RT
   3. Perform QC:
      1. In a suitable container for thin-layer chromatography, add EtOAc to a height of 0.5 cm and close the container
      2. On the Whatman® no.1 paper strip, draw a mark 1 cm from the bottom and a line 6 cm above the mark
      3. Spot 2 µL of radiotracer solution on the mark and immediately place the strip in the TLC container. It is not necessary to dry the spot before developing the strip.
      4. Develop the strip until the solvent front reaches the 6 cm line
      5. Read the strip using a linear radioTLC scanner with a β^+^ probe, or a phosphorimager system.
      6. Integrate the areas under the peaks at R_f_ = 0 (AUC_0_) and R_f_ = 0.9-1 (AUC_1_). There should be no intermediate peak. The radiochemical yield of [^89^Zr]Zr-oxine is calculated as:

$$RCY (\%)=100\times\frac{{AUC}_{0}}{{AUC}_{0}+{AUC}_{1}}$$

      7. RCY ≥ 85% is acceptable.
2. Cell isolation and radiolabelling:
   1. Isolate cells according to standard protocols for the desired cell type, removing any protein from the medium by washing the cells with PBS or 0.9% NaCl. For WBCs, follow the guidelines from Roca *et al*. [1]
   2. Resuspend the cells in PBS or 0.9% NaCl, at 5-10$\times$10^6^ cells/mL in a 50 mL centrifuge tube.
      NB: using cell concentrations lower than 5$\times$10^6^ cells/mL may result in reduced labelling efficiency. Using cells concentrations higher than 10$\times$10^6^ cells/mL is possible, depending on cell type.
   3. Add the [^89^Zr]Zr-oxine solution to the cells in a volumetric ratio (radiotracer:cell suspension) of 1:30 or lower.
   4. Gently swirl the cell suspension every 5 min over a period of 15 min
   5. To the radiolabelled cell suspension, add PBS or 0.9% NaCl to the 50 mL mark and gently resuspend by inverting the tube several times.
   6. Remove unincorporated ^89^Zr by centrifuging the cell suspension for 10 min at 300 *g*
   7. Transfer the supernatant into a separate tube and measure the activity (A_SN_)
   8. Resuspend the cell pellet in the appropriate medium for subsequent use and measure the activity of the cells (A_P_).
   9. Determine the radiolabelling efficiency (LE%) using the following equation:

$$LE\%=100\times\frac{A_{P}}{A_{P}+A_{SN}}$$

NB: labelling efficiency can depend on the cell type.

[1] M. Roca, E.F.J. de Vries, F. Jamar, O. Israel, A. Signore, Guidelines for the labelling of leucocytes with 111In-oxine, Eur. J. Nucl. Med. Mol. Imaging. 37 (2010) 835–841. https://doi.org/10.1007/s00259-010-1393-5.

**Table S1. Calculation of the amount of unchelated oxine in the [^89^Zr]Zr-oxine and [^111^In]In-oxine formulations**

|  | **[^89^Zr]Zr-oxine** | **[^111^In]In-oxine** |
| --- | --- | --- |
| Specific activity of radiometal | > 45.6 mCi/μg | > 50 mCi/μg^#^ |
| Isotopic mass of radionuclide | 88.9 | 110.9 |
| Molar activity | > 150 MBq/nmol^*^ | > 205 MBq/nmol |
| Radiochemical purity (determined by TLC) | 90% | 90% |
| Activity of chelated radiometal in 20 MBq batch of radiometal-oxine | 18 MBq | 18 MBq |
| Amount of chelated radiometal in 20 MBq batch | 18/150 = 0.120 nmol | 18/205 = 0.088 nmol |
| Molecular weight of oxine (8-hydroxyquinoline) | 145.16 | |
| Number of oxinate anions in complex | 4 | 3 |
| Amount of complexed oxine | 0.480 nmol | 0.264 nmol |
| Mass of complexed oxine | 69.68 ng | 38.32 ng |
| Total mass of oxine in formulation | 50 μg | |
| **Percentage of complexed oxine** | 0.14 % | 0.077% |
| **Percentage of “free” oxine in formulation** | 99.86% | 99.93% |

^*^manufacturer specification for zirconium-89
^#^from European Pharmacopoeia v5.0 monograph

The calculations above demonstrate that the majority of the oxine in each formulation is not in a complex with zirconium-89 or indium-111, and that potential differences in “free” oxine content between the formulation are too minor to explain differences in labelling efficiency.

**
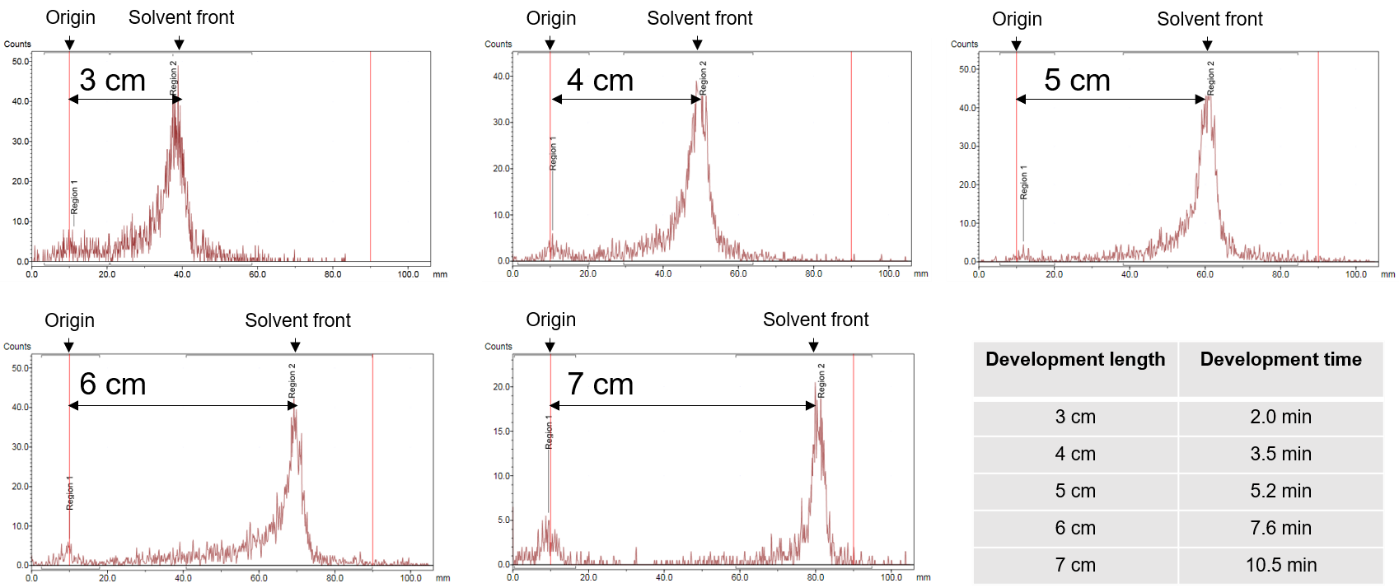
**

**Figure S1: determination of optimal TLC strip length for QC of [^89^Zr]Zr-oxine.** Representative radioTLC chromatograms of [^89^Zr]Zr-oxine on Whatman no.1 paper, with 100% ethyl acetate as mobile phase. Distances in cm were measured from the spotting point (1 cm from the bottom of the strip). The strips were removed from the mobile phase when the solvent front reached the indicated length. A development length of 6 cm offers a good compromise between resolution and speed.

**
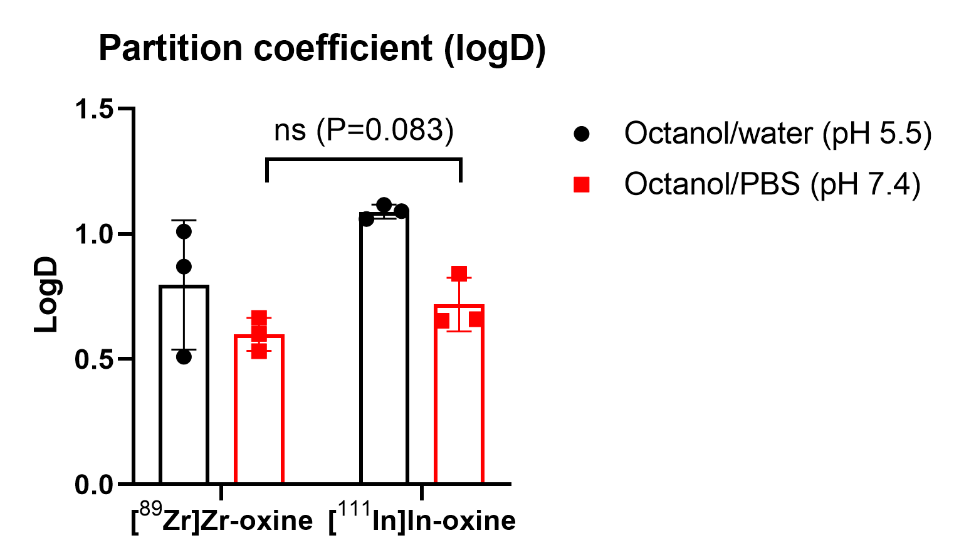
**

**Figure S2: partition coefficients of [^89^Zr]Zr-oxine and [^111^In]In-oxine.** [^89^Zr]Zr-oxine and [^111^In]In-oxine were added to mixtures of octanol and water or octanol and PBS and vortexed. After separation of phases, the activity present in 100 µL of each phase was determined by gamma-counting. The logD values were obtained by taking the log_10_ of the ratio of activity in the octanol layer divided by activity in the aqueous phase. Bars represent the mean±SD of n = 3 separate experiments. Student’s paired *t‑*test was used to compare means. LogD values above 0 indicate that the compounds are both lipophilic. There was no significant difference between the logD values of [^89^Zr]Zr-oxine and [^111^In]In-oxine.


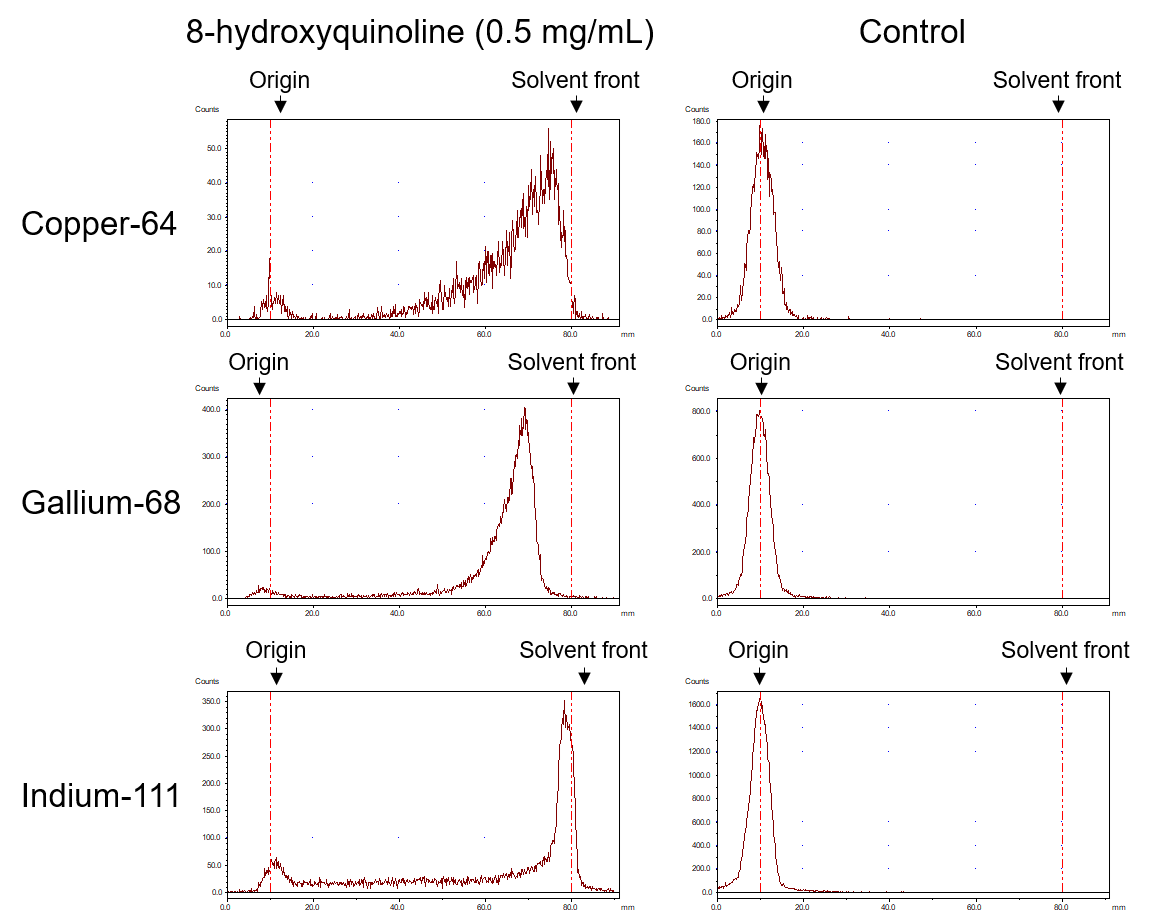


**Figure S3: use of the kit formulation to form lipophilic oxine complexes of copper-64, gallium-68 and indium-111.** Radionuclides were added in the typical form in which they are supplied from cyclotron target extraction or generator elution. To 100 µL of the kit formulation of 8-hydroxyquinoline, copper‑64 in 1-2 M HCl, gallium-68 in 0.1 M HCl or indium-111 in 0.1 M HCl were added and left for 10 min at RT. Controls represent stock solutions of the respective radionuclides as provided by the manufacturers or eluted from the generators. RadioTLCs were performed as described in the Methods.


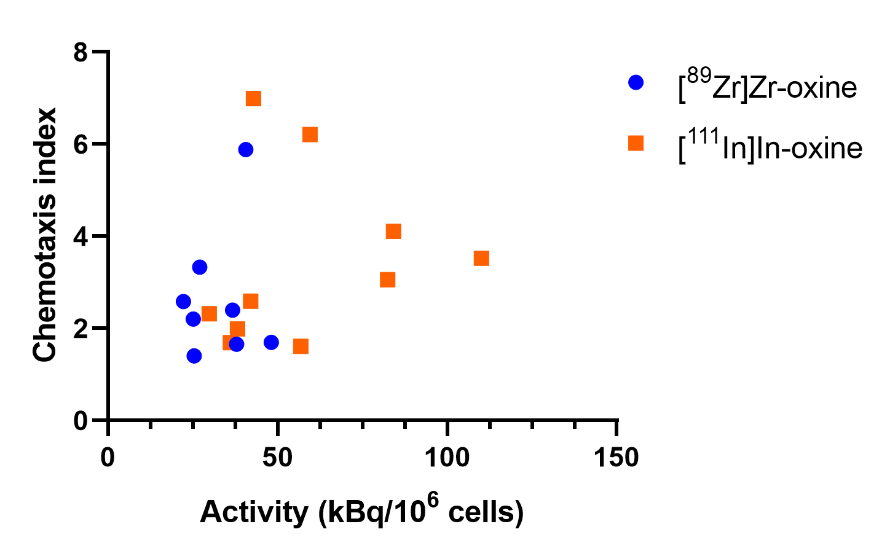


**Figure S4: WBC chemotaxis as a function of cell radioactivity.** Chemotactic index of radiolabelled leukocytes plotted against the amount of activity per cell after radiolabelling with [^89^Zr]Zr-oxine (n = 8) or [^111^In]In-oxine (n = 10). Each point represents an individual donor. No trend was apparent relating chemotaxis index and activity per cell for either radiotracer.


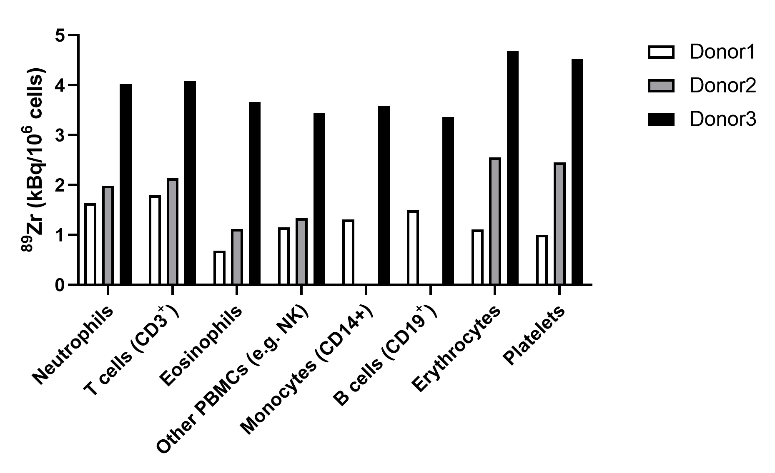


**Figure S5: uptake of ^89^Zr in WBC populations after labelling of mixed WBC with [^89^Zr]Zr-oxine.** Radiolabelled WBC were sorted by FACS (>10000 cells/population) and gamma-counted. Each bar represents cells from an individual donor. The numbers of CD14^+^ and CD19^+^ events in the sample from donor 2 were not sufficient for analysis.


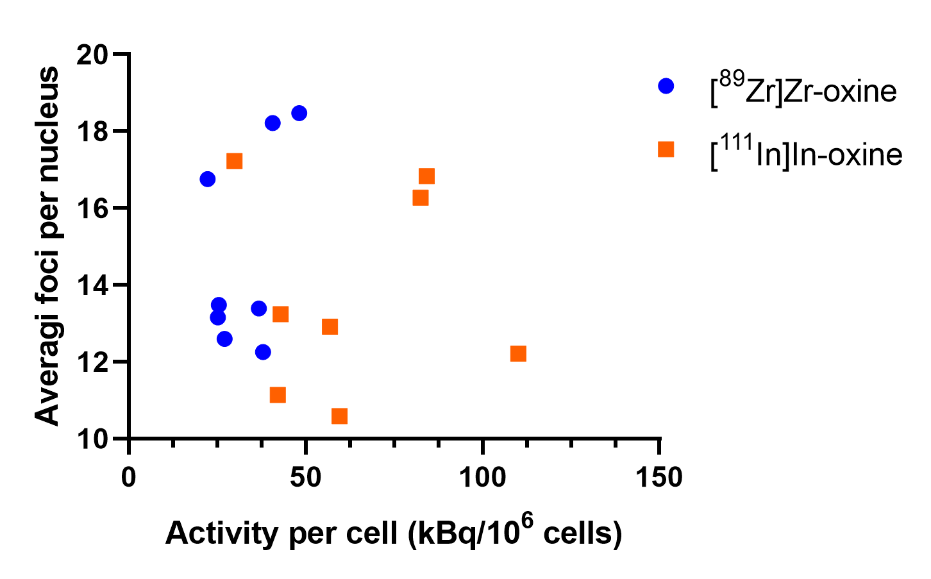


**Figure S6: comparison of DNA damage induced by [^89^Zr]Zr-oxine and [^111^In]In-oxine in WBC.** Average number of γ-H2AX foci per nucleus (at least 30 nuclei analysed per sample), plotted against the amount of activity per cell after radiolabelling. Each point represents an individual donor (n = 8 per radiotracer). No trend was apparent relating the number of foci per nucleus and activity per cell for either radiotracer.
